# Quantifying, displaying and accounting for heterogeneity in the meta-analysis of RCTs using standard and generalised *Q *statistics

**DOI:** 10.1186/1471-2288-11-41

**Published:** 2011-04-07

**Authors:** Jack Bowden, Jayne F Tierney, Andrew J Copas, Sarah Burdett

**Affiliations:** 1 MRC Clinical Trials Unit, 222 Euston Road, London NW1 2DA, UK; 2 MRC Biostatistics Unit, Robinson Way, Cambridge, CB2 0SR, UK

## Abstract

**Background:**

Clinical researchers have often preferred to use a fixed effects model for the primary interpretation of a meta-analysis. Heterogeneity is usually assessed via the well known *Q *and *I*^2^ statistics, along with the random effects estimate they imply. In recent years, alternative methods for quantifying heterogeneity have been proposed, that are based on a 'generalised' *Q *statistic.

**Methods:**

We review 18 IPD meta-analyses of RCTs into treatments for cancer, in order to quantify the amount of heterogeneity present and also to discuss practical methods for explaining heterogeneity.

**Results:**

Differing results were obtained when the standard *Q *and *I*^2^ statistics were used to test for the presence of heterogeneity. The two meta-analyses with the largest amount of heterogeneity were investigated further, and on inspection the straightforward application of a random effects model was not deemed appropriate. Compared to the standard *Q *statistic, the generalised *Q *statistic provided a more accurate platform for estimating the amount of heterogeneity in the 18 meta-analyses.

**Conclusions:**

Explaining heterogeneity via the pre-specification of trial subgroups, graphical diagnostic tools and sensitivity analyses produced a more desirable outcome than an automatic application of the random effects model. Generalised *Q *statistic methods for quantifying and adjusting for heterogeneity should be incorporated as standard into statistical software. Software is provided to help achieve this aim.

## Background

Meta-analysis provides a way of quantitatively synthesising the results of medical studies or trials that target a particular research question. As shown in a 2005 review of the clinical research literature [1], it is still most common to meta-analyse results across clinical studies using the inverse variance approach, to yield a 'fixed' or 'common' effect estimate. By obtaining individual patient data (IPD) from all trials in a meta-analysis, some aspects of clinical heterogeneity can be minimised through data cleaning [2]. However, regardless of whether the meta-analysis is based on IPD or aggregate data, substantial statistical heterogeneity between studies may still remain.

Cochran's *Q *statistic has long been used to assess statistical heterogeneity in meta-analysis. When *Q *is larger than its expected value *E*[*Q*] under the null hypothesis of no heterogeneity, the difference *Q *- *E*[*Q*] can be used to furnish the most popular estimate of the heterogeneity parameter, using the DerSimonian and Laird method [3]. Higgins and Thompson's *I*^2^ statistic [4,5] is also a simple function of *Q *and quantifies the proportion of total variation that is between trial heterogeneity. Unlike *Q*, *I*^2^ is designed to be independent of the number of trials constituting the meta-analysis and independent of the outcome's scale, so it can easily be compared across meta-analyses. It is now reported as standard, with or without Cochran's *Q*.

The presence of significant and substantial heterogeneity demands some form of action. Ideally, after exploration of the data, heterogeneity can be explained by variation in the constituent trial's characteristics. If this is not possible then some may feel a meta-analysis inappropriate altogether, whereas some would opt for fitting a random effects model to the data instead. There is no accepted rule for deciding on when a move from a fixed to a random effects model is the right course of action [6]. Clearly, all other things being equal, the larger the magnitude of the heterogeneity the stronger the case for a shift. However, as the amount of heterogeneity increases, so too does the potential impact of moving from one model to the other. Thus, with increasingly diverging interpretations, it is sometimes very difficult to make a satisfactory decision on which model to choose, or indeed whether to pool the trials in a meta-analysis at all.

In *Methods *we review the standard approach to meta-analysis and heterogeneity quantification based on the *Q *statistic. We then introduce a similar approach based on a 'generalised Q' statistic that has recently been proposed. In *Results *we analyse the summary data from 18 separate IPD meta-analyses to see whether the original conclusions could have been sensitive to the choice of fixed or random effects model. A more in-depth analysis is then conducted on the two meta-analyses with the largest observed heterogeneity. The 18 meta-analysis are then used to illustrate the relative performance of the standard and generalised *Q *statistics in measuring the extent of heterogeneity present. Finally, in *Discussion *and *Conclusions *we review the issues raised and offer recommendations for the future quantification and reporting of heterogeneity in meta-analysis.

### The data

The MRC Clinical Trials Unit has carried out systematic reviews and IPD meta-analyses, predominantly in cancer since 1991. Their common primary aim has been to assess whether treatment interventions have improved patient survival. Specific areas of focus include cancers of the brain, lung, cervix, ovaries and bladder. Table 1 shows the summary statistics of 18 such IPD meta-analyses [7-17]. The usefulness of these meta-analyses is that they all pre-specified subgroup analyses by trial and patient characteristics in order to explain potential heterogeneity. For illustration these analyses are done ignoring any pre-specified groupings and are only with respect to the primary outcome of overall survival. A two-stage approach was taken for each meta-analysis treatment comparison. That is, fixed effect hazard ratio estimates were calculated for each trial using the log rank method, [18], these estimates were then combined using fixed and random effects models in the same manner as for aggregate data. The meta-analyses differed in terms of their size (from 5-19 studies), their fixed effect hazard ratio effect estimate (0.65-1.20) and their heterogeneity (Q statistic p-values from 1.97 × 10^-5^ to 0.99 and *I*^2^ from 0 to 75%).

**Table 1:** The summary statistics for 18 meta-analyses carried out by the MAG.

Meta-analysis	# trials	Q, P-value	**(%)**	Fixed Effect HR (CI) P-value
cervix 1 [15]	18	44.48, 0.00	62	1.05 (0.93-1.19) 0.39
cervix 2 [17]	18	20.83 0.23	18	0.76 (0.67-0.85) 0.00
cervix 3 [15]	5	9.18, 0.06	56	0.65 (0.53-0.80) 0.00
bladder 1 [14]	9	7.27, 0.51	0	0.91 (0.83-1.01) 0.08
bladder 2 [16]	6	2.25, 0.81	0	0.75 (0.60-0.96) 0.02
nsclc 1 [8]	17	28.98, 0.02	45	1.04 (0.96-1.12) 0.33
nsclc 2 [8]	7	3.63, 0.73	0	0.98 (0.83-1.14) 0.76
nsclc 3 [8]	25	22.32, 0.56	0	0.90 (0.83-0.97) 0.01
nsclc 4 [8]	11	39.63, 0.00	75	0.84 (0.74-0.95) 0.01
ovarian 1 [7]	19	21.92, 0.24	18	0.98 (0.91-1.06) 0.69
ovarian 2 [7]	11	12.83, 0.23	22	0.93 (0.83-1.05) 0.23
ovarian 3 [10]	9	14.78, 0.06	46	0.88 (0.79-0.98) 0.02
ovarian 4 [10]	9	10.35, 0.24	23	0.91 (0.80-1.05) 0.21
ovarian 5 [10]	12	2.57, 1.00	0	1.02 (0.93-1.12) 0.66
port [11]	9	13.06, 0.11	39	1.21 (1.08-1.34) 0.00
sarcoma [9]	14	11.80, 0.54	0	0.89 (0.76-1.03) 0.12
oeso [12]	6	10.37, 0.07	52	0.89 (0.78-1.01) 0.06
glioma [13]	12	13.29, 0.27	17	0.85 (0.78-0.92) 0.00

## Methods

Consider a meta-analysis of *M *studies. When study *i *out of *M*'s effect estimate - denoted by  - is assumed to be normally distributed with a known variance , then one can think of the study estimates as centered around a common mean parameter *θ *as in formula (1):(1)

The *ϵ*_*i *_term relates to the precision of study *i*'s estimate, and is assumed to follow a *N* (0, ) distribution.

The *u_i _*term is assumed to have zero mean and a variance of *τ*^2^; it is included to represent potential between trial heterogeneity. When *τ*^2^ equals 0 all studies provide an estimate of the same mean parameter *θ *. Under the assumption that *τ*^2^ is 0 the fixed effect (FE) estimate, associated variance and assumed asymptotic distribution can be obtained:(2)

where *W_i _= *1/ is study *i'*s precision.

### Heterogeneity quantification using the standard *Q*-statistic

If the fixed effects assumption is true then Cochran's statistic:(3)

should follow, asymptotically, a *χ*^2^ distribution, with expected value equal to *M - *1. However, if *τ*^2^ is non zero so that there is a degree of heterogeneity among the trials, study *i *provides an estimate of *θ *+ *u_i _*and the expected value of *Q *equals(4)

where , and is referred to as the 'typical' within study variance.

The most commonly applied estimate of *τ*^2^ is due to DerSimonian and Laird [3]. This simply replaces *E*[*Q*] in formula (4) with its observed value in formula (3) and solves *τ*^2^ to give what we term . This estimate is truncated to zero if negative and then used to provide re-weighted overall mean estimate (and variance *V_RE_*) by replacing *W_i _*in (2) with . The 'RE' subscript denotes 'random effects'. This method has become synonymous with random effects meta-analysis, because of its ease of use - it does not require statistical maximisation software and does not impose constraints on the distribution of the random effects *u_i _*[19]. Furthermore,  can be used to furnish the most popular measure of the extent of heterogeneity - Higgins and Thompson's *I*^2^ value [4,5] - since

when *Q *> *M *- 1.

From a philosophical perspective, fixed effect and random effects estimates target very different quantities. Fixed effect models estimate the weighted mean of the study estimates, whereas random effects models estimate the mean of a distribution from which the study estimates were sampled. However, if model (1) is correct and we are additionally willing to assume that the *u_i _*terms are independent of the *ϵ*_*i *_terms, then they should both provide estimates of the same parameter *θ*. Another consequence of this independence assumption is that the individual study estimates  should be independent of the *ϵ*_*i *_terms, and hence we do not expect the magnitude of the effect estimate to be correlated with its precision.

### Heterogeneity quantification using a 'generalised' *Q*-statistic

 is very easy to calculate but may itself be a misleading estimate of the true heterogeneity present. More sophisticated likelihood-based methods - such as 'REML' [20], or Bayesian methods using MCMC [21] - may be preferred, but are more computationally demanding to calculate and also impose distributional assumptions on the random effects. Recently, a method has been championed that combines some of the computational simplicity of the DerSimonian and Laird method, with the rigor and accuracy of likelihood based approaches. DerSimonian and Kacker [22] (and others [23-25]) have noted that a generalisation of the *Q *statistic in equation (3) can be written as:(5)

where  and where  is also calculated from equation (2) by replacing *W_i _*with . Like the standard *Q *statistic in equation (3), this also follows a  distribution under the null hypothesis of no heterogeneity. Paule and Mandel [23] (PM) and DerSimonian and Kacker [22] propose to estimate *τ*^2 ^by iterating equation (5) until *Q*(*τ*^2^) equals its expected value of *M*-1; this estimate will be referred to as . DerSimonian and Kacker recommend using  since it is still very easy obtain, is guaranteed to have at most one solution and provides a more accurate estimate of *τ*^2 ^that closely mirrors both the REML estimate and the generalized Bayes estimate [24], which are both much harder quantities to obtain computationally.

Viechtbauer [25] suggests that equation (5) can additionally be used to provide an *α*-level confidence set for , by finding the values of *τ*^2^ that equate *Q*(*τ*^2^) with the *α*/2th and 1- *α*/2th percentiles of the  distribution. He showed that this method performed very well in a simulation study that evaluated its coverage properties compared to a range of other methods - such as Biggerstaff and Tweedie [26] and Sidik and Jonkman [27] - primarily because it is based on an exact *χ*^2^ distribution, rather than a distributional approximation.

A criticism one might therefore have of *I*^2^ is that its standard definition is intertwined with the commonly applied DerSimonian and Laird estimate . A generalised *I*^2^ statistic, say , could easily be defined for a meta-analysis with typical within study variance *s*^2^ as

for any estimate of the between study variance . From now on we will refer to Inconsistency statistics specifically utilising the DL method as  and those specifically utilising the PM method as . The term *I*^2^ will be reserved for discussing the general concept of Inconsistency.

### Reference intervals for  and 

Since the Inconsistency statistic is a data derived estimate, it is possible to plot a confidence (or 'reference') interval around it to highlight its inherent uncertainty. Higgins and Thompson [4] recommend basing reference intervals for  using the variance of the related 'H' measure, since they are simple functions of one another. This involves using one of two formulae depending on the value of *Q *relative to *M*. The lower bound of these intervals, if negative, is curtailed at zero. We will calculate (1-*α*) level reference intervals for  for each meta-analysis as(6)

where  and  represent the values of *τ*^2^ equating *Q*(*τ*^2^) to the lower *α*/2 and upper 1-*α*/2 percentiles of the relevant *χ*^2^ distribution.

## Results

### A standard *Q*-statistic analysis

**Figure 1:**
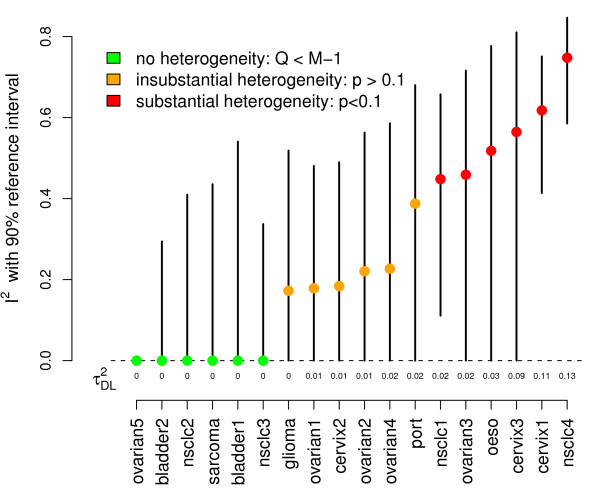
**The **** statistic (with 90% reference intervals) from each study**; ** also shown.**

Figure 1 shows the  statistics (plus 90% reference intervals) for the 18 meta-analyses in Table 1. The  estimates for each study are also shown. In order to compliment the Inconsistency reference intervals, the 18 trials are coloured according to their assigned *Q *statistic status; green for no heterogeneity (*Q *<*M *- 1), orange for non-zero but insubstantial heterogeneity (p-value > 0.1) and red for substantial heterogeneity (p-value < 0.1). We can not plot a meaningful  reference interval for the 'Ovarian 5' meta-analysis [10]. This had the smallest estimated  value of -3.28. Whilst the point estimate is curtailed to 0, the upper 90% reference limit calculated from the exact value is still negative.

One could use the reference intervals around  to directly test for the presence of heterogeneity, as apposed to *Q*; a strategy suggested by Medina et. al. [28]. From Figure 1 we see that only 3 out of the 7 meta-analyses with significant *Q *statistics produced significant  statistics at the 10% level. Since *Q *and  are so closely related it is perhaps surprising to some reviewers that such differing conclusions could arise.

**Figure 2:**
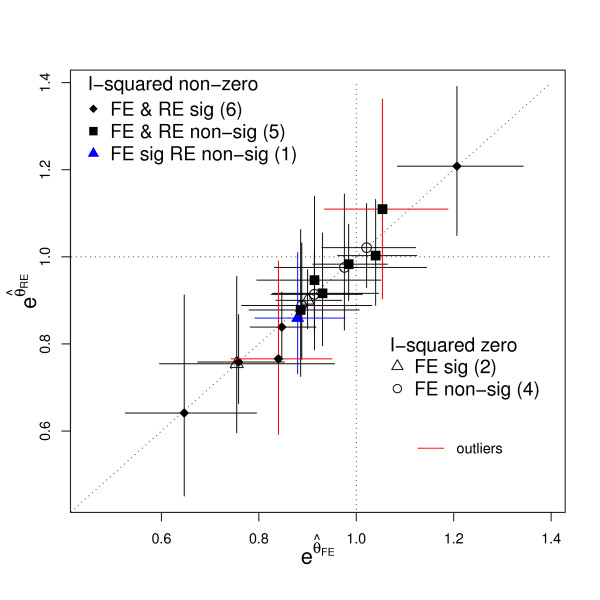
**A comparison of the hazard ratio estimates (with 95% CI) obtained for the 18 meta-analyses in Table 1 under fixed and random effects (DL) models**.

Figure 2 plots the hazard ratio estimates and associated 95% confidence intervals obtained for each trial under the fixed effect and random effects models. The random effects estimates are obtained via the two-stage DL method previously described to highlight their differences. The correlation between the two hazard ratio measures across trials is naturally high. Six out of the 18 meta-analyses exhibit no heterogeneity at all. That is, the Q-statistic was less than its expected value - providing an estimate for  of zero and an  of zero. Of these six, 2 meta-analyses showed a significant fixed effect estimate at the 5% level and 4 did not. From the remaning 12 meta-analyses with an  > 0 and so with distinct fixed and random effects estimates, the clinical interpretation of the overall results is generally similar by either approach. For example, only one out of the 18 meta-analyses - Ovarian 3 [10] - do we see the fixed and random effects estimates with p-values either side of 0.05, that may lead some to interpret the meta-analysis differently.

From Figure 1, the two meta-analyses with the most apparent statistical heterogeneity were NSCLC 4 [8] and Cervix 1 [15]. They also exhibit the most marked differences between their fixed and random effects estimates, as highlighted by large deviations from the diagonal - shown in red in Figure 2. These two meta-analyses are now discussed further, in order to demonstrate how we chose to investigate these heterogeneous data sets.

#### The NSCLC 4 meta-analysis

This meta-analysis compared the effectiveness of supportive care plus chemotherapy versus supportive care alone for patients with advanced non-small cell lung cancer. The fixed effect hazard ratio estimate of 0.84 suggests a substantial and highly significant benefit from the addition of chemotherapy with a p-value for a null effect of 0.005. The random effects model estimate of 0.77 suggested an even more extreme benefit of chemotherapy. However, such was the magnitude of heterogeneity detected - as revealed by an *I*^2^ of 75% - this estimate is attributed much less certainty, with a p-value of 0.04.

**Figure 3:**
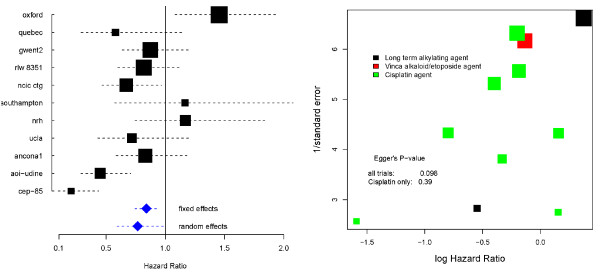
Left: Forest plot of the NSCLC 4 meta-analysis; Right: funnel plot of the NSCLC 4 meta-analysis.

On a closer inspection of these data, the size of the study appears to be correlated with its estimated effect - indeed the largest study (*oxford*) and the smallest study (*CEP*-85) cover the complete range of all the estimates. Figure 3 (right) shows a funnel plot [29] of the same data to highlight this more clearly. If the independence assumption in equation (1) holds, then we would expect the plot to be symmetrical around the mean estimate. Although funnel plot asymmetry is not necessarily indicative of dissemination bias (or 'small study' effects), the prevailing, uncontroversial view is that unbiased study dissemination is more likely to occur for larger studies than for smaller studies, and it is certainly one possible explanation for what we see here. Egger's regression [30] - which can be thought of as a very general test for dissemination bias [31] - provides some evidence for a higher than average correlation between effect size and precision (p-value 0.098). This correlation is the reason for the large difference between the estimates  and . As the contributing trials spanned 30 years, the types of chemotherapy used varied considerably and consequently the pre-specified main analysis sub-divided trials into chemotherapy categories. This was indeed helpful in resolving the issue before any further, more subjective analyses were attempted. Of the 11 relevant trials identified, it was found that 2 trials used long term alkylating agents, 1 trial used a Vinca alkaloid/etoposide agent and 8 used a Cisplatin based regimen. The results of this subgroup analysis are shown against the results across all trials in Table 2. Among the 8 Cisplatin trials we saw a slight decrease in the estimated magnitude of heterogeneity with  down to 68%. The homogeneity p-value from the standard *Q*-statistic was also far less significant, but is arguably due in some degree to a loss of power resulting from splitting the data. The main effect was however a clear reduction in the amount of funnel plot asymmetry (Egger's regression p-value 0.38) and consequently much better agreement between fixed and random effects model estimates.

**Table 2:** Subgroup analyses for the two examples.

Trial Group	# trials	**Q, P-value, ****(%)**	Fixed Effect HR (CI) P-value	Random Effects HR (CI) P-value
NSCLC data				
all	11	39.6 (1.97e-05) 74.8	0.84 (0.74-0.95) 5.42e-03	0.77 (0.59-0.99) 0.042
Cisplatin	8	22.2 (2.34e-03) 68.5	0.73 (0.63-0.85) 6.63e-05	0.70 (0.53-0.93) 0.014
*Q_int _*= 39.62 - (22.20 + 8.72) = 8.70 (p = 0.003)				
all*	11		0.84 (0.61-1.16) 0.21	
Cervix data				
>14 days	11	12.76 (0.24) 22	1.25, (1.07,1.46) 0.005	1.27 (1.06,1.53) 0.0099
≤ 14 days	7	20.74 (0.002) 71	0.83, (0.69,1.00) 0.046	0.87 (0.60,1.25) 0.44
*Q_int _*= 44.48 - (12.76 + 20.74) = 10.98 (p = 9e-04)				

*Q*_*int *_denotes the subgroup interaction test statistic. * denotes the Henmi-Copas analysis: which puts a 'random effects' confidence interval around the fixed effects estimate.

#### The Cervix 1 meta-analysis

**Figure 4:**
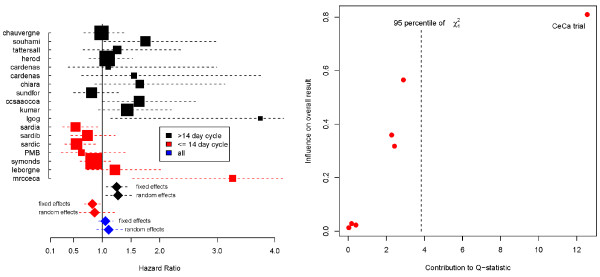
Left: Funnel plot of the Cervical cancer trial data; Right: Baujat plot showing, for the ≤ 14 day subset of trials, the influence of each trial on the overall *Q *statistic and fixed effect estimate.

This meta-analysis compared neoadjuvant chemotherapy plus local treatment versus local treatment alone for patients with Cervical cancer. Both the fixed and random effect estimates indicated a slight benefit in the experimental treatment but neither estimate is Significant (Table 1). Significant heterogeneity was present in the 18 trials with a reported  of 62%. In the original research process, clinical advice influenced a pre-planned decision to split the studies into two groups, depending on whether chemotherapy was given in cycles lasting longer than, or less than, 14 days. The results for these subgroups are shown in Figure 4 (left) and Table 2. No evidence of heterogeneity was apparent in the trials using longer chemotherapy cycles. Furthermore, for this subgroup both the fixed effect and random effects estimates suggest a highly Significant and substantial detrimental effect of neoadjuvant chemotherapy.

Significant heterogeneity persisted in the results of trials using shorter chemotherapy cycles. The fixed effect result suggested a modest benefit from short cycle chemotherapy, whereas the random effects model suggested less of an effect and a much wider confidence interval overlapping the null effect of 1. However our conclusions were also guided by a sensitivity analysis of the shorter duration trials, excluding the *MRC CeCa *trial. Figure 4 (right) shows a Baujat plot [32,33] of the data; on the horizontal axis is the contribution of each study to the overall *Q *statistic in equation (3), on the vertical axis is the difference between the fixed effect estimate  with and without each study, standardised by the total variance of the fixed effect estimate without that study. If the fixed effects model is correct, each point's horizontal component should be approximately  distributed. The *CeCa *trial is way out on its own, whereas the other trials all fall within the 95th percentile of this distribution. Thus the total heterogeneity present is very much a product of this single trial. Furthermore, the *CeCa *trial's large vertical component shows that its inclusion Significantly alters the fixed effects estimate too. Excluding the *CeCa *trial gave a fixed-effect result still favouring short cycle chemotherapy (HR = 0.76, 95%CI = 0.62-0.92) and heterogeneity was much reduced. Repeating the sensitivity analysis using a random effects model gave very similar results (HR = 0.75,95%CI = 0.58-0.95).

### A generalised-*Q* analysis

**Figure 5:**
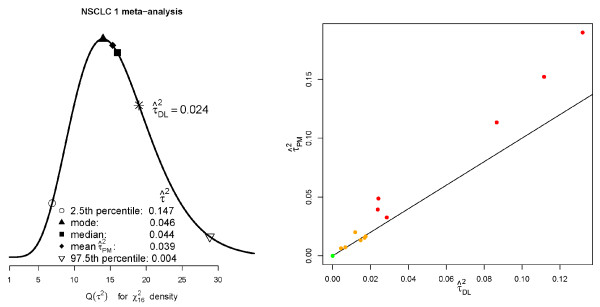
Left: Point estimates (and lower/upper bounds) for the between trial variance parameter of the NSCLC1 meta-analysis, using the Q-profile approach; Right:  versus  for all 18 meta-analyses.

Figure 5 (left) shows the generalised estimation procedure enacted on the NSCLC1 meta-analysis. From Figure 1 this meta-analyses, along with NSCLC 4 and Cervix 1, exhibited such strong heterogeneity that its 90%  reference interval did not contain 0. It contains 17 studies and hence an estimate for  is obtained by equating *Q*(*τ*^2^) to a *χ*^2^ statistic with 16 degrees of freedom. We plot the  estimate corresponding to its mean of 16. We also plot two more solutions that have not been considered so far; setting *Q*(*τ*^2^) to the median or 50th percentile of the *χ*^2^ distribution (which seems consistent with the approach of Viechtbauer), and also to the mode of *M *- 2. The spread of these solutions, determined by the skewness of the *χ*^2^ distribution makes clear how the uncertainty as to the value of *τ*^2^ is heavily dependent on the number of studies. The DerSimonian and Laird estimate  is equal to 0.024. When this is plugged into *Q*(*τ*^2^) it would equate to a *χ*^2^ statistic with approximately 19 degrees of freedom. Viechtbauer's 95% confidence set for *τ*^2^ is (0.004-0.147). Figure 5 (right) shows, for all 18 meta-analyses, how the estimates for  based on the generalised *Q *statistic compared to the original estimates - , the original traffic light colour markings are retained.

**Figure 6:**
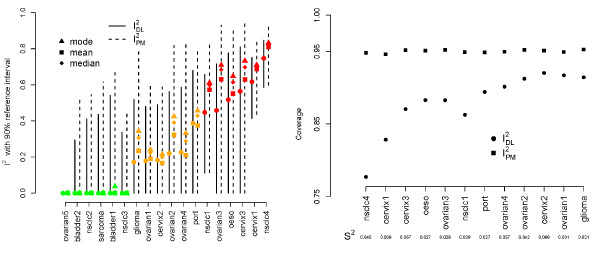
**Left: point estimates and 90% reference intervals for **** and **** estimates; Right: Coverage of 95% reference intervals for the  and  estimates.**

Figure 6 (left) plots Higgins and Thompson's  plus 90% reference interval (as in Figure 1) versus equivalent reference intervals for  - as shown in equation (6). We additionally highlight the mean, median and modal values of , to indicate the spread of even these central measures.

### A simulation study

The point estimates and confidence intervals for  differ from the original  - in particular the confidence intervals for  are noticeably wider. In order to see if this extra width truly reflected the uncertainty in the estimation of , or instead if it was over-conservative, we conducted twelve simulation studies, each one based on the characteristics of a meta-analysis which exhibited some heterogeneity (from 'Glioma' to 'NSCLC 4'). From each one we took the number of studies *M*, within study variances *σ*^2^ and the DL heterogeneity estimate . For meta-analysis *j*, *j *= 1, ..., 12, we then simulated 10,000 new meta-analyses of *M_j _*study estimates  for *i *= 1, ... *M_j _*. The choice of *θ *= 0 is clearly unimportant. Since the within study variances and the true *τ*^2^ values were held fixed, the true value of *I*^2^ stayed fixed at the original value reported in Table 1, and . We then calculated the proportion of 95% reference intervals for  and  that contained the true value. Figure 6 (left) shows the results. Higgins and Thompson's  reference interval appears to exhibit sub-optimum coverage, which is especially clear when the true value of *I*^2^ is large. Reference intervals for  based on equation (6) appear to well maintain the desired coverage across all 12 simulation scenarios.

## Discussion

### NSCLC 4 and Cervix 1

As mentioned in *Methods*, in the presence of heterogeneity we still expect fixed and random effects estimates to be targeting a single quantity. However, in *Results *the two meta-analyses with the largest heterogeneity also showed that largest empirical differences between  and . The NSCLC 4 data was a good example of this, being the meta-analysis with the largest outward heterogeneity, but with also clear funnel plot asymmetry. If we had been ignorant as to the type of chemotherapy used in each study, and therefore had no way of explaining the heterogeneity, we would perhaps have considered applying a random effects model, despite suspecting small study effects. Random effects estimation in this context can start to look considerably less attractive, because  gives *more *(rather than less) relative weight to the smaller studies than  since for any study *i*, *W_i _*≥ , a fact first highlighted by Greenland [34]. This has lead some to propose bias adjustment procedures to counteract small study effects [35-37]. Henmi and Copas [38] have recently advocated an interesting compromise; to use the fixed effects point estimate  - that is robust to small study effects - but surrounded by a confidence interval derived under the random effects model. As shown in (Table 2), when applied to all studies in the NSCLC 4 meta-analysis this puts a 95% confidence interval of (0.61-1.16) around  = 0.84, with an associate p-value of 0.21, bringing the treatment's benefit severely into doubt. Fortunately, we were able to plausibly explain most of the asymmetry present by the differing types of chemotherapy regimens used, providing a much more useful answer with added clinical insight.

For the Cervix meta-data, stratifying the trials by chemotherapy cycle duration helped to partially explain the heterogeneity. Again, in doing so it raised interesting clinical questions about the effective treatment of this cancer. The remaining heterogeneity present in the short cycle chemotherapy trials was removed by excluding an outlying study in a sensitivity analysis, guided by the results of a Baujat plot. Throwing data away is generally frowned upon by statisticians, and more sophisticated methods for incorporating so called 'outliers' have been proposed [39]. However, for small outlying studies this strategy is clearly a convenient and effective option. We could find no explanation for the extreme effect found by the *CeCa *trial in its design or patient population, but it is perhaps worth noting that, along with the *PMG *and *LGOG *trials, its results were never published in a peer reviewed journal. Clearly, one of the advantages of a meta-analysis is to bring together the totality of evidence, including especially trials whose results were not fully disseminated in the past. We do not know if the extreme results observed specifically in the *CeCa *and *LGOG *trials influenced their original non-publication, but it is certainly worrying that the overall picture of evidence is far easier to interpret in their absence.

### Standard or Generalised *Q* statistic?

In *Methods and Results *we described and demonstrated the use of meta-analytical techniques based on the generalised *Q *statistic. Are these worth using? As can be seen from Figure 5 (right), whenever  is zero so is . For non-zero values  is generally greater than , the difference between the two appears to increase as the magnitude of the heterogeneity increases. This suggests that when a substantial amount of heterogeneity is present,  may be systematically underestimating it because a one-iteration formula is not sufficient to arrive at an estimate near the truth. This underestimation does not effect in any meaningful way the estimate for *θ*. Across the 18 meta-analyses, the random effects estimates for  based on  and  were very similar (and are therefore not shown) since the overall mean estimate is fairly insensitive to small changes in *τ*^2^[25,40]. However, the variance of , *V_RE_*, and *I*^2^ are far more sensitive to changes in *τ*^2^ and hence accurate estimation *is *important for these quantities.

## Conclusions

In this paper we have restricted our focus to the estimation of the meta-analytical quantities *τ*^2^, *I*^2^ and the overall mean parameter *θ*, as well as providing confidence intervals for the latter two. We note that this does not reflect the state-of-the-art in what can estimated via a random effects meta-analysis; one can for instance also estimate trial level effect parameters (*θ *+ *u_i_*), predict the likely effects of future studies and test hypotheses relating to these additional parameters [19]. With this in mind, we make the following tentative conclusions.

The actual magnitude of the estimate *τ*^2^ is often overlooked as a heterogeneity measure [41], and in keeping with modern developments the Dersimonian and Laird estimate is no longer considered to be the best choice [22,24]. We recommend using the PM estimate for *τ*^2^ - and by extension the  it implies - since it is still very easy to calculate, but shares much of the accuracy and rigor of more complex methods. Van der Tweel and Bollen [42] use the PM method to estimate the overall random effects mean *θ_RE _*and heterogeneity parameter within the context of a sequential meta-analysis, but appear to stick with the original  for other aspects of their analysis. We recommend that practitioners additionally make use of the PM estimate in the Inconsistency measure . R code to estimate , *θ_RE _*and  (with confidence intervals) is provided below.

An *I*^2^ of over 75% has traditionally been considered as indicating a high level of inconsistency, *I*^2^'s of above 50% as moderate and *I*^2^'s of below 25% as low. It is tempting to consider a random effects model when the *I*^2^ is high. However, the range of the reference intervals shown in Figure 6 (left) highlights the considerable uncertainty around this measure. The recently updated Cochrane handbook [6] now gives overlapping rather than mutually exclusive regions for low, moderate and high heterogeneity, but when the heterogeneity is measured with as much uncertainty as in the Cervix 3 meta-analysis (90% reference intervals for  of 0% to 93%) any categorisation feels dubious. Inconsistency intervals based on the  statistic will generally be wider than those based on the standard  measure but is a more accurate reflection of the uncertainty present. These findings are based on a fairly large simulation study for widely varying *τ*^2^, typical within study variance *s*^2^ and trial number *M*. Although the simulated data were normally distributed, we do not think the conclusions would have changed if the study effects had been drawn from a more non-standard distribution. By plotting  at the lower and upper reference levels, as well at a spread of more central measures such as the mean, median and mode, one can easily and effectively convey this uncertainty to the analyst. For a comprehensive comparison of methods for estimating the heterogeneity parameter *τ*^2^ see Biggerstaff and Tweedie [26] or Viechtbauer [25].

In the presence of heterogeneity, the naive and automatic application of the random effects model has been widely criticised. It is sensible to conduct a further investigation the data [34,43,44], but this may not lead to the identification of any explanatory factors. If unexplained heterogeneity also leads to large differences between the fixed and random effects estimates, there is the obvious prospect that conflicting clinical interpretations could arise. When funnel plot asymmetry is the predominant cause of this, *I*^2^ statistics have a less meaningful interpretation. For this reason Rücker et. al [37] have recently proposed an alternative 'G' statistic, that expresses the inconsistency between studies after this asymmetry has been accounted for (through a bias correction for small study effects). As demonstrated on the NSCLC meta-analysis, the Henmi-Copas method combining a fixed effects estimate with a 'random effects' confidence interval provides an alternative way of dealing with funnel plot asymmetry without making an explicit bias correction. Both the approaches of Rücker et. al. and Henmi and Copas appear to offer sensible and practical solutions to this problem, and merit further investigation.

## R code

This code calculates point estimates and *α*-level confidence intervals for ,  and , given the estimated effect sizes *y *within study standard errors *s *and desired type I error *Alpha*. This code is based on the algorithm suggested by DerSimonian and Kacker [22].

PM = function(y = y, s = s, Alpha = 0.1){

K = length(y) ; df = k -1 ; sig = qnorm(1-Alpha/2)

low = qchisq((Alpha/2), df) ; up = qchisq(1-(Alpha/2), df)

med = qchisq(0.5, df) ; mn = df ; mode = df-1

Quant = c(low, mode, mn, med, up) ; L = length(Quant)

Tausq = NULL ; Isq = NULL

CI = matrix(nrow = L, ncol = 2) ;MU = NULL

v = 1/s^2 ; sum.v = sum(v) ; typS = sum(v*(k-1))/(sum.v^2 - sum(v^2))

for(j in 1:L){

tausq = 0 ; F = 1 ;TAUsq = NULL

while(F>0){

TAUsq = c(TAUsq, tausq)

w = 1/(s^2+tausq) ; sum.w = sum(w) ; w2 = w^2

yW = sum(y*w)/sum.w ; Q1 = sum(w*(y-yW)^2)

Q2 = sum(w2*(y-yW)^2) ; F = Q1-Quant[j]

Ftau = max(F,0) ; delta = F/Q2

tausq = tausq + delta

}

MU[j] = yW ; V = 1/sum(w)

Tausq[j] = max(tausq,0) ; Isq[j] = Tausq[j]/(Tausq[j]+typS)

CI[j,] = yW + sig*c(-1,1) *sqrt(V)

}

return(list(tausq = Tausq, muhat = MU, Isq = Isq, CI = CI, quant = Quant))

}

## List of Abbreviations

IPD: Individual Patient Data; FE: fixed effect; DL: DerSimonian and Laird; RE: Random effects; PM: Paule-Mandel.

## Competing interests

The authors declare that they have no competing interests.

## Authors' contributions

JFT and SB produced an early version of this paper. JB substantially revised the paper to bring it to its current form. AJC and JFT provided invaluable advice to JB during this process. All authors read and approved the final manuscript.

## Authors' information

JB is a biostatistician working within the London and Cambridge MRC hubs for trials methodology research. JFT is the head of the Meta-analysis group at the MRC Clinical Trials Unit (CTU). AJC is a senior statistician within the MRC CTU and also a senior lecturer in medical statistics at University College, London. SB is a systematic reviewer at the CTU, working within the Meta-analysis group.

## Pre-publication history

The pre-publication history for this paper can be accessed here:

http://www.biomedcentral.com/1471-2288/11/41/prepub
